# The Impact of Age at First Lambing on Milk Yield and Lactation Length in a Population of Istrian Sheep under Semi-Intensive Management

**DOI:** 10.3390/ani11061604

**Published:** 2021-05-29

**Authors:** Ante Kasap, Jelena Ramljak, Boro Mioč, Valentino Držaić, Ivan Širić, Darko Jurković, Marija Špehar

**Affiliations:** 1 Faculty of Agriculture, University of Zagreb, Svetošimunska 25, 10000 Zagreb, Croatia; jramljak@agr.hr (J.R.); bmioc@agr.hr (B.M.); vdrzaic@agr.hr (V.D.); isiric@agr.hr (I.Š.); 2 Croatian Agency for Agriculture and Food, Svetošimunska 25, 10000 Zagreb, Croatia; darko.jurkovic@hapih.hr (D.J.); marija.spehar@hapih.hr (M.Š.)

**Keywords:** sheep, age, first lambing, milk

## Abstract

**Simple Summary:**

The impact of age at first lambing on dairy traits has been poorly investigated in sheep, especially in semi-intensive husbandry systems. Insufficient information on this issue, especially scientifically proven, leads to many speculations among breeders. The aim of this study was to examine the impact of age at first lambing on days in milk, daily milk yield, and total milk yield. The study was conducted on field data routinely collected in a population of Istrian sheep bred under selection. It was determined that the prolongation of the first mating of lamb ewes to the second year of life is not beneficial to milk production. In conclusion, ewes reared under semi-intensive dairy orientated systems could be successfully bred in their first year of life in order to reduce their unproductive non-milking phase.

**Abstract:**

This study aimed to examine the impact of ewe’s age at first lambing (AFL) on days in milk (DIM), average daily milk yield (DMY), and total milk yield (TMY). Symmetrical bimodal distribution of AFL enabled classification of maidens in those mated in the first (47%) or second year of life (53%). After accounting for all available sources of phenotypic variability with the linear mixed model for repeated records, it was estimated that AFL had a statistically significant effect only on DIM (p < 0.001). The litter size had a significant effect only on TMY (*p* < 0.001), while the effect of the parity was significant for all the examined traits (*p* < 0.001). The results of the study suggest that prolongation of age at first mating to the second year of life is not justified in dairy-orientated sheep farms. However, more evidence on this issue is needed for generalization, especially considering some other traits that can impact profitability of dual-purpose sheep farms (reproduction traits, growth rate of lambs, etc.).

## 1. Introduction

Dairy-orientated sheep husbandry systems, dominantly present in the Mediterranean basin, mainly rely on local breeds well-adapted to the specific environment and seasonal availability of pastures [1]. The majority of systems “tagged” as a dairy are actually a dual purpose, with a substantial portion of incomes provided from lamb’s meat [2]. In France, Greece, Italy, and Spain (representatives of the dairy sheep sector), the average milk yield varies from low to medium (85–216 L/ewe/lactation) [3]. A similar range of milk yields is present in Croatia where dairy sheep farming relies on three foreign (Lacaune, East-Friesian, and Travnik pramenka) and two autochthonous breeds (Pag sheep and Istrian sheep).

The Istrian sheep is a dual-purpose breed originating from the Istrian peninsula whose formation officially started in 1771. Many foreign breeds (rams) were involved in creation of the breed: Gentile di Puglia, Bergamasca, Southdown, Merinolandschaf, Merino, Awassi, and East-Friesian [4,5]. In total, 1632 individuals are currently included in the national selection program [6]. BLUP has been used as selection tool for about a decade now [7] with a test-day repeatability animal model [8]. The transition to a single-step genomic BLUP is underway.

Planning of the breeding in flocks, especially in seasonally sensitive systems, presents a challenge for farmers since both economical and biological considerations need to be carefully balanced [9,10]. The optimum age at first lambing in dairy sheep heavily relies on husbandry system, local dairy facilities policies, inherent characteristics of the breed, etc. However, with all the information available, both from the practice and science, it is still somewhat difficult to determine the optimal age for lamb ewes to be bred. The breeding of ewe lambs as soon as possible without repercussions on their productivity is an efficient way of making dairy sheep operations more productive by reducing the unproductive phase of the females. On the other hand, breeding lamb ewes at a very young age poses risk for incomplete ewe’s body development and soundness. 

Conversely to the abundant information of optimal age at first rearing in beef [11,12,13] and dairy [14,15,16,17,18,19] cattle, information on this issue for dairy sheep is limited. Very few systematically conducted studies on this have been published so far for dairy sheep, and none of them was related to semi-intensive (pasture-based) rearing. Gootwine and Pollott [20,21] found that breeding Awassi and Assaf ewe lambs later in life led to increased milk production in their first lactation, but they concluded that it was not economically justified. Hernandez et al. [22] in a study conducted on Lacaune breed reported the optimal age at first lambing is 13–15 months. However, both of the above-mentioned studies were conducted in intensive management systems. Absence of scientific evidence leads to many speculations on this issue, thus the aim of this study was to examine the effect of age at first lambing on some dairy characteristics of Istrian sheep reared in a semi-intensive (pasture-based) breeding environment.

## 2. Materials and Methods

### 2.1. Animals

The study was conducted using field phenotypic records collected on 1891 Istrian ewes born between 2009 and 2018 in 101 flocks. The ewes under study had been performance tested for the purpose of the national selection program on dairy traits. All the data used in the study were provided by the Croatian Ministry of Agriculture. The ewes had been raised in a semi-intensive husbandry system. Natural pastures had been the main source of food all year round, except in the couple of cold winter months (depending on the year) when hay represented the major component of the rations (1.5–2 kg/day). In addition to forages (pasture and hay), ewes were fed with concentrates (corn or corn/barley) in the last two months of gestation and during lactation (mainly while being milked or after milking, usually 200–500 g/day).

### 2.2. Data

**Figure 1:**
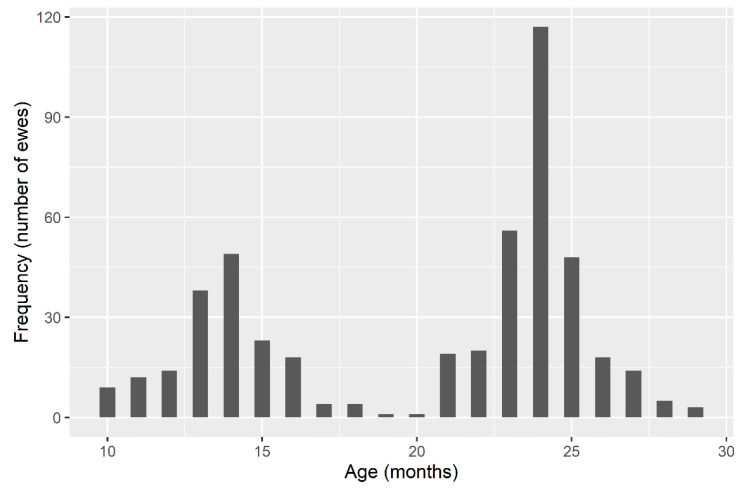
Distribution of age at first lambing (AFL) of Istrian ewes.

In total, 4725 records for each trait (days in milk (DIM), total milk yield in the milking period (TMY), and average daily milk yield (DMY)) were used in the analysis. Test–day records were collected monthly in accordance with the regular alternate scheme (morning/evening system) based on ICAR rules [23]. The TMY per lactation was estimated using the Fleischmann’s method [24] from test-day records for all ewes that had at least three milk recordings within lactation. The first recording was conducted between the 5th and 30th day after weaning and thereafter for approximately 30 days (28–34) until the end of lactation (i.e., when daily milk secretion fell below 0.2 kg). The age at first lambing (AFL) was available for all ewes under study. The distribution of AFL had two peaks centered at 14 and 24 months of age (Figure 1). Such distribution of AFL enabled classification of the ewes on those mated for the first time in the first year of life (AFL1 = 10–17 months, *n* = 889), and those mated for the first time in the second year of life (AFL2 = 18–29 months, *n* = 1002).

**Figure 2:**
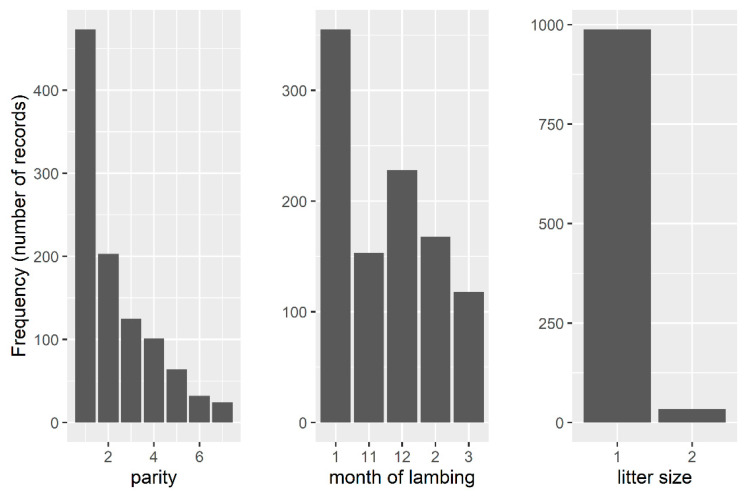
Frequency distribution of phenotypes by parity, month of lambing, and litter size.

Prior to the estimation of the effect of AFL on the examined traits, a thorough analysis of distribution of phenotypic records by parity, litter size, and lambing season was conducted. Several adjustments of the original data were performed in order to retain all available data in the analysis as follows: multiple births were set to Class 2+, parities after 7th were set to Cass 7+, October lambings were set to November lambings, while April lambings were set to March lambings. The frequency distribution of phenotypes by parity, litter size, and lambing season after these adjustments is shown in Figure 2.

### 2.3. Statistical Analysis

The inferential statistical analysis is suited to the unbalanced measurement experimental design with repeated measurements. Linear mixed models (LMM) represent an extension of simple linear models to allow accounting for both fixed and random effects. These models are particularly useful when there is non-independence in the data [25]. Accounting for random variability in the model was performed to correct the standard errors for non-independence of the data and not to explore variability within the classes of random variables. Prior to the analysis, the significance level α was set to 0.05. After testing for the significance of the available fixed effects and comparing performances of several different statistical models, the following mixed linear model (Equation (1)) was used in the analysis of the total milk yield (TMY) and daily milk yield (DMY):

(1) $$y_{ijklmn}=\mu +b_{1}\left(s_{ijklmn}-\bar{s}\right)+b_{1}\left(m_{ijklmn}-\bar{m}\right)+P_{i}+LS_{j}+AFL_{k}+hym_{l}+a_{m}+e_{ijklmn}$$

where *Y_ijklmn_* is the nth observation and µ is the overall mean in groups *ijklm*. Parity (*P_i_*, *I* = 1,…, 7+), litter size (*LS_j_*, *j* = 1, 2+), and age at first lambing (*AFL_k_*, *k* = 1, 2) were fitted as class, while length of suckling (*s*) and milking (*m*) period as continuous fixed predictors. The herd–year–month of lambing (*hym*) and animal (*a*) were fitted as random effects. The effects length of suckling (*s*) and length of milking (*m*) period were fitted as fixed continuous predictors (linear regressions). It might seem at first that one of these covariates is redundant in the model, but it turned out that both covariates were informative and, moreover, practically uncorrelated (see the paper by Kasap et al. [26] for more details). The reduced statistical model (Equation (2)), without covariates *s* and *m*, was used in the analysis of the days in milk (DIM).

(2) $$y_{ijklmn}=\mu +P_{i}+LS_{j}+AFL_{k}+hym_{l}+a_{m}+e_{ijklmn}$$

Separate random effects terms were considered independent. In both models, the covariance structure was unstructured. We did not impose any constraints to the values, so each variance and each covariance were estimated uniquely from the data which provided the best possible fit (very close to what the data reflected).

The statistical analysis was conducted in R programing environment [27]. Figures were obtained with package “ggplot2” [28]. The package “lme4” [29] was used in estimation of the AFL on the examined traits. The R function “lmer()” is described in detail in [30]. Estimation of the least square means (LSM) and their standard errors was obtained with the package “emmeans” [31].

## 3. Results

### 3.1. Descriptive Statistics

**Figure 3:**
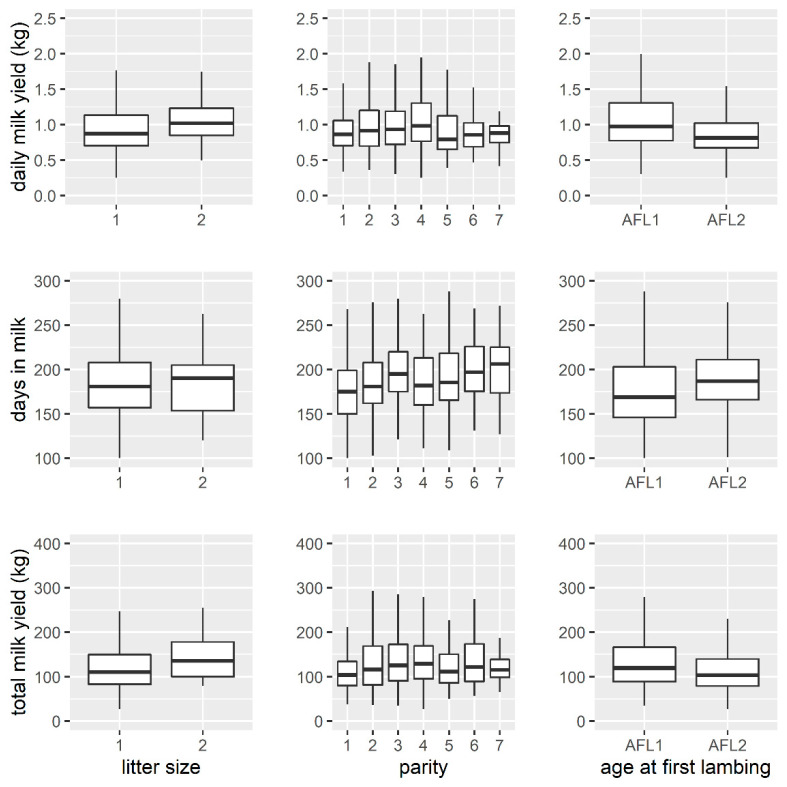
Distributions of daily milk yield (DMY), days in milk (DIM), and total milk yield (TMY), classified by litter size, parity, and age at first lambing.

Distributions of the analyzed traits classified by litter size, parity, and AFL are shown in Figure 3. On average, multiple-bearing ewes had longer lactation, produced more milk in the milking period, and had higher DMY. DIM consistently increased with parity while TMY and DMY only up to the third parity and then decreased. Ewes mated for the first time in the second year of life (AFL2) had higher DIM but lower DMY and TMY. AFL2 showed greater homogeneity in phenotypic expression for all the examined traits. 

### 3.2. Inferential Statistics

The results of the inferential statistical analysis (Table 1) are generally in line with the raw means discussed above (descriptive statistics). The estimated marginal means (LSM) presented in Table 1 were averaged across the levels of the other fixed class effects for the sake of simplicity and better insight into the magnitude of the particular effect. The estimates (LSM) obtained from the same statistical model, but simultaneously taking into account all the fixed class effects, without averaging on the other effects, are presented in Table 2. These results enable comparisons of the estimates across all possible levels of class fixed effects, and they correspond to the summarized estimates in Table 1. Description of the results in this form gives the opportunity to easily get information on expected performances of Istrian sheep by simultaneously considering these three sources of phenotypic variability. 

### 3.3. Age at First Lambing 

The effect of the main interest in this study, AFL, significantly affected only DIM (*p* < 0.001). It was estimated that lactation lasted 14 days longer in AFL1, but without positive impact of AFL on milk yield (both TMY and DMY). In fact, it was estimated that early mated lamb ewes (AFL1) tend to produce negligibly more milk than AFL2. Close inspection of the results (Table 2) revealed that AFL had a negative effect on the milk yield traits (DMY and TMY) and a positive effect on the DIM irrespective of the parity and litter size. 

### 3.4. Litter Size 

The litter size had a positive effect on DMY and TMY and a negative effect on DIM. However, the effect was statistically significant only for TMY (*p* < 0.001). Multiple bearing ewes produced ~10 L milk more in lactation. As determined above for AFL, the estimated effect of parity was consistent across other class fixed effects in the model (Table 2).

### 3.5. Parity

Parity had a significant effect on all the traits in the analysis (*p* < 0.001). Estimates for milk yield (DMY and TMY) reached a maximum in third parity and thereafter decreased, while estimated for DIM increased consistently up to the last class of parity (7^+^). The maximal difference in adjacent estimates was determined between fourth and fifth parity for DMY and TMY and between first and second parity for DIM. 

## 4. Discussion

Semi-intensive dairy orientated sheep breeding systems dominate over intensive dairy systems in many Mediterranean countries, where the majority of European sheep milk is produced. In such conditions, particularly welcomed efforts are those that tend to increase productive potential of the animals with little or no direct investments. The scientific knowledge on the impact of AFL on ewe’s dairy performance is scarce, and there are still many unanswered questions and doubts on this issue, not only among breeders but also among scientists. The question of optimum AFL has been comprehensively addressed by some studies (e.g., [21,22]), but investigated only under intensive management systems where undesired environmental effects can easily be alleviated. However, ewe’s performance in conditions where ewes dominantly graze and receive limited amounts of concentrate (strong impact of natural environmental conditions) might have different outcome. Estimation of this effect is pretty challenging in many non-intensively managed sheep populations, mainly due to a lack of available phenotypic data and all other necessary information on systematic effects. Fortunately, this breed has been in the performance recording system for dairy traits for more than a decade. The availability of a large number of systematically collected phenotypes and other important information gave us the opportunity to get some answers with the appropriate statistical model. The population of Istrian sheep under study exhibited a wide range of AFL (from 10 to 29 months), although with a bimodal distribution with peaks at 14 (47%) and 24 (53%) months. The average DIM was 183 days (52 days of suckling and 131 days of milking period). The average DMY and TMY yield in milking period was 1.08 L (sd = 0.50) and 142.5 L (sd = 80.44), respectively. Classification of the observations by parity and litter size was in accordance with our expectations (based on our own experience and many reported results on this issue from the literature). On the other hand, the results pertaining to the AFL, which was the main goal of the study, only partially match with the previous findings on this issue. The lamb ewes mated in their first year of life (AFL1) had a two weeks shorter lactation period, but DMY and TMY were only marginally affected by the AFL. Actually, it turned out that ewes mated earlier in life (AFL1) had negligibly higher DMY and TMY compared to ewes mated in their second year of life (irrespective of the parity). However, the difference between the groups of AFL was not statistically significant for the both of the analyzed traits. Contrary to these results, Pollott and Gootwine [21] in their study on Assaf breed with the AFL range between 10 and 28 months found that AFL did not affect DIM, but it considerably affected TMY. They reported that older ewe lambs at the first parity produced more milk in the first lactation, but less milk in second lactation (the estimated regression coefficients of TMY on AFP were 3.7 L/month and −1.82 L/month, respectively). Hernandez et al. [22] reported similar results for Lacaune breed. They also found a positive relationship between AFL and the first lactation TMY and negative relationship between AFL and the second lactation TMY. In addition to the plenty of important findings from their study, it is also worth mentioning here that ewes mated for the first time later in life reached their maximum TMY in their first lactation. This is in accordance with the previous reports for Lacaune [32], as well as for some other sheep breeds (e.g., Latxa, [33]). The authors argued that lambing at younger age probably had lower first lactation yields because of directing greater amounts of energy towards growth during their first lactation compared to ewes lambing at older age when they reached a mature size. Our results are not completely consistent with those reported above because our lamb ewes mated later in life (AFL2) did not exhibit better performance in the first lactation (TMY and DMY). In addition, regardless of the AFL, the ewes at the first parity did not outperform those in later parities. However, the results pertaining to the impact of the AFL on the later lactations correspond with their reports to substantial extent. Our experience and the results of this study suggest that breeding lamb ewes early in life (AFL1) has no detrimental impact on the milk yield, neither in the first nor in the later parities. Based on these results, as well as those reported earlier, we hereby argue that prolongation of the mating (until the second year of life) is not justified by itself if there are no particular detectable obstacles (e.g., poor body development, poor condition, or some kind of unsolved health problems). The authors above also concluded their work on this issue in the same manner because it was found that the milk gains in the first lactation accompanied by increased AFL could not compensate losses in the future lactations and shorter lifetime production. We hereby argue that the results of this study nicely reflect the impact of the AFL on DIM, TMY, and DMY in the population of Istrian sheep reared in a semi-intensive husbandry system. The results should serve as scientific evidence for future breeding activities in this population and some other populations reared in the Mediterranean semi-intensive dairy orientated system.

## 5. Conclusions

The results of this study suggest that breeding maiden ewes in the first year of life does not diminish their lifetime dairy performances. To reduce the unproductive phase of the animals, breeding should not be prolonged to another year if soundness, body development, and condition are not compromised. 

Although the applied statistical approach partially accounted for the additive genetic effects in revealing the direction and magnitude of the target effect (AFL), more evidence is needed for generalization. 

Analysis under the framework of the genetic animal model could provide more evidence on this issue, particularly in the context of the single step genomic BLUP. Extension of this research is advised to take into account some other important traits that can affect the overall success dairy-orientated sheep facilities, such as fertility, growth rate of lambs, etc. 

## Figures and Tables

**Table 1:** Estimated effects of age at first lambing (AFL), litter size, and parity on daily milk yield (DMY), total milk yield (TMY), and days in milk (DIM). The estimates are averaged across the levels of other class fixed effects in the statistical model.

		DMY (kg)			TMY (kg)			DIM (Days)		
	n	LSM	SE	Sign.	LSM	SE	Sign.	LSM	SE	Sign.
AFL										
AFL1	2573	1.10	0.02	NS	144.44	4.89	NS	185.90	2.14	***
AFL2	2152	1.04	0.02		142.92	4.95		199.26	2.20	
Litter size										
1	4443	1.05	0.01	NS	138.57	4.67	***	193.02	1.91	NS
2+	282	1.09	0.02		148.79	5.40		192.15	2.71	
Parity										
1	1715	1.08	0.02	***	144.46	4.83	***	171.50	2.13	***
2	1034	1.13	0.02		154.34	4.91		183.39	2.21	
3	770	1.13	0.02		154.11	4.98		191.41	2.29	
4	545	1.10	0.02		151.05	5.10		193.90	2.42	
5	310	1.04	0.02		138.41	5.41		197.36	2.74	
6	193	1.00	0.03		130.41	5.79		205.22	3.11	
7+	158	1.00	0.03		132.97	6.08		205.30	3.35	

AFL, age at first lambing; Sign., significance level; NS, not significant; *** = *p* < 0.001; LSM, least square means; SE, standard error.

**Table 2:** Estimates of the possible levels of class fixed effects from the model for daily milk yield (DMY), total milk yield (TMY), and days in milk (DIM).

Parity	AFL	Litter Size	DMY		TMY		DIM	
			LSM	SE	LSM	SE	LSM	SE
1	AFL1	1	1.07	0.04	140.11	4.76	165.25	2.00
2	AFL1	1	1.13	0.04	149.99	4.85	177.14	2.12
3	AFL1	1	1.13	0.04	149.76	4.91	185.17	2.19
4	AFL1	1	1.11	0.04	146.70	5.03	187.66	2.32
5	AFL1	1	1.03	0.04	134.06	5.31	191.12	2.63
6	AFL1	1	0.98	0.04	126.06	5.69	198.97	3.01
7	AFL1	1	0.98	0.04	128.62	5.97	199.05	3.25
1	AFL2	1	1.05	0.03	138.59	4.73	178.61	1.98
2	AFL2	1	1.12	0.04	148.47	4.84	190.50	2.11
3	AFL2	1	1.11	0.04	148.24	4.95	198.52	2.22
4	AFL2	1	1.09	0.04	145.18	5.09	201.01	2.38
5	AFL2	1	1.02	0.04	132.54	5.40	204.48	2.71
6	AFL2	1	0.96	0.04	124.54	5.80	212.33	3.09
7	AFL2	1	0.96	0.04	127.10	6.08	212.41	3.34
1	AFL1	2	1.10	0.04	150.33	5.53	164.39	2.83
2	AFL1	2	1.16	0.04	160.21	5.56	176.28	2.87
3	AFL1	2	1.16	0.04	159.98	5.59	184.30	2.91
4	AFL1	2	1.14	0.04	156.92	5.68	186.79	2.99
5	AFL1	2	1.06	0.04	144.28	5.96	190.25	3.25
6	AFL1	2	1.01	0.05	136.28	6.30	198.11	3.56
7	AFL1	2	1.01	0.05	138.84	6.57	198.19	3.77
1	AFL2	2	1.08	0.04	148.80	5.50	177.74	2.82
2	AFL2	2	1.15	0.04	158.69	5.55	189.63	2.86
3	AFL2	2	1.15	0.04	158.46	5.63	197.66	2.93
4	AFL2	2	1.12	0.04	155.40	5.74	200.15	3.03
5	AFL2	2	1.05	0.04	142.76	6.04	203.61	3.31
6	AFL2	2	0.99	0.05	134.76	6.40	211.46	3.63
7	AFL2	2	0.99	0.05	137.32	6.67	211.54	3.85

AFL, age at first lambing; LSM, least square means; SE, standard error.

## Data Availability

The data presented in this study are available on request from the corresponding author. The data are not publicly available to preserve privacy of the data.
